# Probiotic lactic acid bacterial strains MG4693 and MG5474 alleviate atopic dermatitis by enhancing intestinal barrier integrity and modulating the immune system

**DOI:** 10.29219/fnr.v70.13890

**Published:** 2026-08-18

**Authors:** Wonchan Yoon, Jeong-Yong Park, Soo-Im Choi, Byoung-Kook Kim, Ji Yeon Lee

**Affiliations:** MEDIOGEN, Co., Ltd., Chungju-si, Republic of Korea

**Keywords:** atopic dermatitis, probiotics, gut–skin axis, Th2 cytokines

## Abstract

**Background:**

Atopic dermatitis (AD) is a chronic inflammatory skin disease characterized by barrier dysfunction and a Type 2 T helper cell-dominant immune response. Probiotics have emerged as promising candidates for the management of AD by modulating intestinal and systemic immune responses.

**Objective:**

This study aimed to investigate the effects of *Lacticaseibacillus paracasei* MG4693 and *Lactococcus lactis* MG5474 on 2,4-dinitrochlorobenzene-induced AD mice.

**Design:**

AD was induced in Bagg Albino/c (BALB/c) mice by repeated topical 2,4-dinitrochlorobenzene applications, followed by the daily oral administration of MG4693 or MG5474 for 5 weeks. Skin lesions and scratching behavior in the dorsal and ear skin were evaluated. Epidermal thickness, immune cell infiltration, and levels of thymic stromal lymphopoietin (TSLP) and filaggrin were assessed by histology and immunohistochemistry. Flow cytometry was used to analyze T- and B-cell populations in lymph nodes. Intestinal barrier integrity was concurrently evaluated to investigate the involvement of the gut–skin axis.

**Results:**

Both strains significantly alleviated AD symptoms, reducing epidermal hyperplasia, eosinophil and mast cell infiltration, and TSLP expression while restoring filaggrin. In addition, these strains modulated T- and B-cell populations in the lymph nodes, indicating an immunomodulatory effect.

**Discussion:**

Amelioration of AD symptoms appears to be mediated by enhanced intestinal barrier integrity and attenuation of Type 2 T helper cell-mediated inflammatory responses.

**Conclusion:**

MG4693 and MG5474 attenuated AD pathology by improving intestinal barrier function and modulating systemic immune responses. They demonstrated potential as probiotic candidates to manage AD by targeting the gut–skin axis.

## Popular scientific summary

- Alleviation of the atopic dermatitis with barriers and immune dysfunction using probiotic lactic acid bacterial strains MG4693 and MG5474 was examined in a 2,4-dinitrochlorobenzene (DNCB)-induced disease mouse model.
- Probiotic supplementation reduced dorsal and ear skin lesions, scratching behavior, and epidermal thickening.
- MG4693 and MG5474 reduced thymic stromal lymphopoietin and Th2 cytokine expression and modulated lymph node immune cell populations, suggesting systemic immunoregulatory effects.
- MG4693 and MG5474 also regulated intestinal barrier-associated genes, increasing the expression of claudin-1, occludin, and zonula occludens-1 (ZO-1).

Atopic dermatitis (AD) is a prevalent chronic inflammatory skin disease that affects individuals of all ages but exhibits a particularly high frequency during early childhood (1). AD is marked by severe pruritus and recurrent eczematous lesions on visible areas, such as the hands and ears, which substantially impair the patient’s quality of life (2). AD is often referred to as ‘eczema’; however, it is a distinct immunological disorder with a multifactorial etiology (3). Its pathogenesis involves a complex interplay of factors, including disruption of the skin barrier, immune dysregulation, genetic predisposition, and environmental exposure (4).

Recent research indicates that increasing global industrialization and associated environmental pollution have contributed to the rising incidence of AD, particularly in highly urbanized and rapidly developing regions (5). Patients with AD typically exhibit impaired skin barrier function, as evidenced by increased transepidermal water loss and elevated skin surface pH (6). These alterations are closely associated with disease severity and may facilitate allergen penetration of the skin barrier and microbial colonization (7). Current treatments for AD include corticosteroids, immunosuppressants, bioactive agents, and Janus kinase (JAK) inhibitors. Although these treatments are effective, they cause side effects such as skin atrophy, conjunctivitis, and systemic infection, as well as an increased risk of developing malignancy (8–11). Therefore, there is growing interest in developing therapeutic strategies for AD that effectively control inflammation while minimizing adverse side effects.

The human gut is a primary immune system-associated organ that harbors approximately 70% of the body’s immune cells and plays a vital role in maintaining immune homeostasis (12). The interaction among the gut epithelium, commensal microbiota, and the mucosal immune system regulates local and systemic immune responses, thereby directly impacting extra-intestinal immune regulation (13). Growing evidence suggests that immune-mediated skin diseases, such as AD, may stem from disturbances in the gut microbiota composition or from interactions with the host immune system (14). Bidirectional communication between the gut and skin, known as the gut–skin axis, may be mediated by microbe-derived metabolites, systemic cytokines, and mucosal immune cells (15).

Probiotics are conventionally defined as live microorganisms that, when delivered in adequate amounts, confer health benefits to the host (16). In addition to modulating the gut microbial composition, probiotics produce metabolites, including short-chain fatty acids, which support the intestinal barrier integrity, maintain the balance between systemic and local immune responses, and suppress inflammation-related processes (17, 18). These barrier-strengthening and immunomodulatory effects are particularly relevant to AD, a condition characterized by skin barrier dysfunction, Type 2 T helper cell (Th2)-mediated immune dysregulation, and the gut microbiota dysbiosis (19).

Given this evidence, targeting the gut–skin axis through immune-related pathways via probiotic supplementation may represent a promising therapeutic approach to AD management. Our previous studies demonstrated that *Lacticaseibacillus paracasei* MG4693 and *Lactococcus lactis* MG5474 attenuate inflammatory responses by suppressing activation of nuclear factor kappa-light-chain-enhancer of activated B-cells (NF-κB) and mitogen-activated protein kinase (MAPK) signaling pathways in HaCaT cells (20). However, the *in vivo* effects of MG4693 and MG5474 on AD pathophysiology, particularly in relation to systemic immune regulation and the gut–skin axis, remain poorly understood. Therefore, this study investigated whether supplementation with the MG4693 and MG5474 strains could alleviate AD-like symptoms in an *in vivo* model and modulate skin barrier function and immune responses.

## Materials and methods

### Preparation of samples and strain culture

*Lacticaseibacillus paracasei* MG4693, isolated from the human oral cavity (NCBI accession number: OP077096.1), and *L. lactis* MG5474, isolated from fermented foods (NCBI accession number: ON619520.1), were provided by MEDIOGEN Co., Ltd. (Jechon, Republic of Korea). These strains were anaerobically cultured in De Man, Rogosa, and Sharpe broth (BD Biosciences, NJ, USA) in an anaerobic chamber at 37°C. These were identified through *16S rRNA* sequencing (SolGent Co., Ltd., Daejeon, Republic of Korea).

### 2,4-dinitrochlorobenzene (DNCB)-induced AD mouse model

Bagg Albino/c (BALB/c) mice, 5-week-old, male (Central Lab. Animal Co., Ltd., Inchon, Korea), were maintained in a regulated environment: temperature of 22 ± 3°C, humidity level of 50 ± 20%, and an illuminance of 150–300 Lux under a 12 h light/dark cycle. Mice were provided *ad libitum* access to a γ-irradiated diet (1,314 IRR; Altromin Spezialfutter GmbH & Co. KG, Lage, Germany) and tap water. All procedures involving animals were approved by the Central Bio Animal Ethics Committee (Approval number: CBIACUC_23-0468EF).

AD was induced by the topical application of 1% DNCB to the dorsal skin and ears twice weekly for 3 weeks following depilation. After the induction period, AD was maintained by applying 0.5% DNCB to the same sites three times per week during the subsequent 5-week treatment period. Mice were then randomly assigned to four groups (*n* = 10/group): 1) naïve, 2) DNCB, 3) DNCB + MG4693, and 4) DNCB + MG5474. MG4693 and MG5474 were orally administered once daily at 1 × 10^9 colony-forming unit (CFU)/head for 5 weeks, whereas the naïve and DNCB groups received phosphate-buffered saline. All mice were sacrificed at the end of the experimental period.

### Evaluation of the AD score

Disease severity was assessed based on the AD scores, with a slight modification of the previously described method (21). The AD score was assessed once a week during the treatment period by applying the following criteria: 1) erythema/hemorrhage, 2) scarring/dryness, 3) edema, and 4) excoriation/erosion, on a four-point scale: 0 = none, 1 = mild, 2 = moderate, and 3 = severe. Observations were conducted independently by two blinded researchers, and the mean value was analyzed.

### Scratching behavior and ear edema

After the DNCB application, the mice were transferred to new cages and allowed a 1-week adaptation period before observation. To distinguish from grooming, only the behaviors performed with the hind limbs were classified as scratching. Three blinded researchers independently performed observations, and the mean value was calculated. The dorsal skin and ears were photographed, and ear thickness was measured with calipers.

### Plasma, tissue collection, and biochemical parameter analysis

At the end of the experiment, the mice were euthanized, and blood was collected from the abdominal aorta into serum separator tubes, which were then centrifuged to obtain serum. The dorsal skin, axillary lymph nodes (ALN), intestines, and spleens were excised, and organs were weighed. Each extracted tissue was flash-frozen in liquid nitrogen and stored at –80°C until analysis. Commercially available enzyme-linked immunosorbent assay (ELISA) kits were used to measure the serum immunoglobulin E (IgE; Thermo Fisher Scientific Inc., Waltham, MA, USA) and interleukin (IL)-4 (DY404; R&D Systems Inc., Minneapolis, MN, USA) contents according to the manufacturers’ instructions. Absorbance was read on a 450 nm microplate reader (Multiskan SkyHigh, Thermo Fisher Scientific Inc.).

### Measurement of messenger RNA (mRNA) levels of cytokines and tight junction-related genes

Total RNA was extracted from the dorsal skin and intestines using TRIzol reagent, and cDNA was synthesized for quantitative polymerase chain reaction (qPCR) analysis. The expression levels of IL-4, IL-5, IL-13, and interferon (IFN)-γ encoding genes in the skin and tight junction-related zonula occludens-1 (ZO-1), occludin, and claudin encoding genes in the intestine were quantified relative to those of glyceraldehyde 3-phosphate dehydrogenase. All primers were synthesized by Bioneer Co., Ltd. (Daejeon, Republic of Korea).

### Histological analysis

For histological analysis, dorsal skin tissues were fixed in 10% neutral buffered formalin (NBF), sectioned at 3 µm thickness, and stained with toluidine blue, hematoxylin & eosin (H&E), and Congo red. Mast cells were counted in four representative fields of dorsal skin tissue per mouse at 200× magnification. Epidermal thickness in three representative fields was measured at 200× magnification using eXcope image analysis software (eXcope Inc.).

Immunohistochemistry (IHC) staining was performed using an IHC kit (ab64261; Abcam Ltd., Cambridge, UK) and an anti-thymic stromal lymphopoietin (TSLP) antibody (LS-B13715; Lifespan Biosciences Inc., Seattle, WA, USA). Color development employed 3,3′-diaminobenzidine. TSLP expression was quantified as the proportion of positively stained area (%). The mean value per field of view was analyzed.

### Flow cytometry

T-cells (anti-CD3) and B-cells (anti-CD19) in the ALN were quantified employing an Attune™ CytPix™ flow cytometer (Thermo Fisher Scientific Inc.).

### Statistical analysis

Statistical analysis was performed using IBM SPSS Statistics software (version 25, IBM Corp., Armonk, NY, USA). Outliers were identified utilizing the Shapiro–Wilk test and excluded from further analysis. The homogeneity of variance was assessed employing a Levene’s test, followed by a one-way analysis of variance. Post hoc comparisons were conducted using the Bonferroni or Dunnett’s T3 tests for equal or unequal variances, respectively. Statistical significance was set at *P* < 0.05.

## Results

### Effect of MG4693 and MG5474 treatment on AD symptoms in DNCB-induced AD mice

**Fig. 1:**
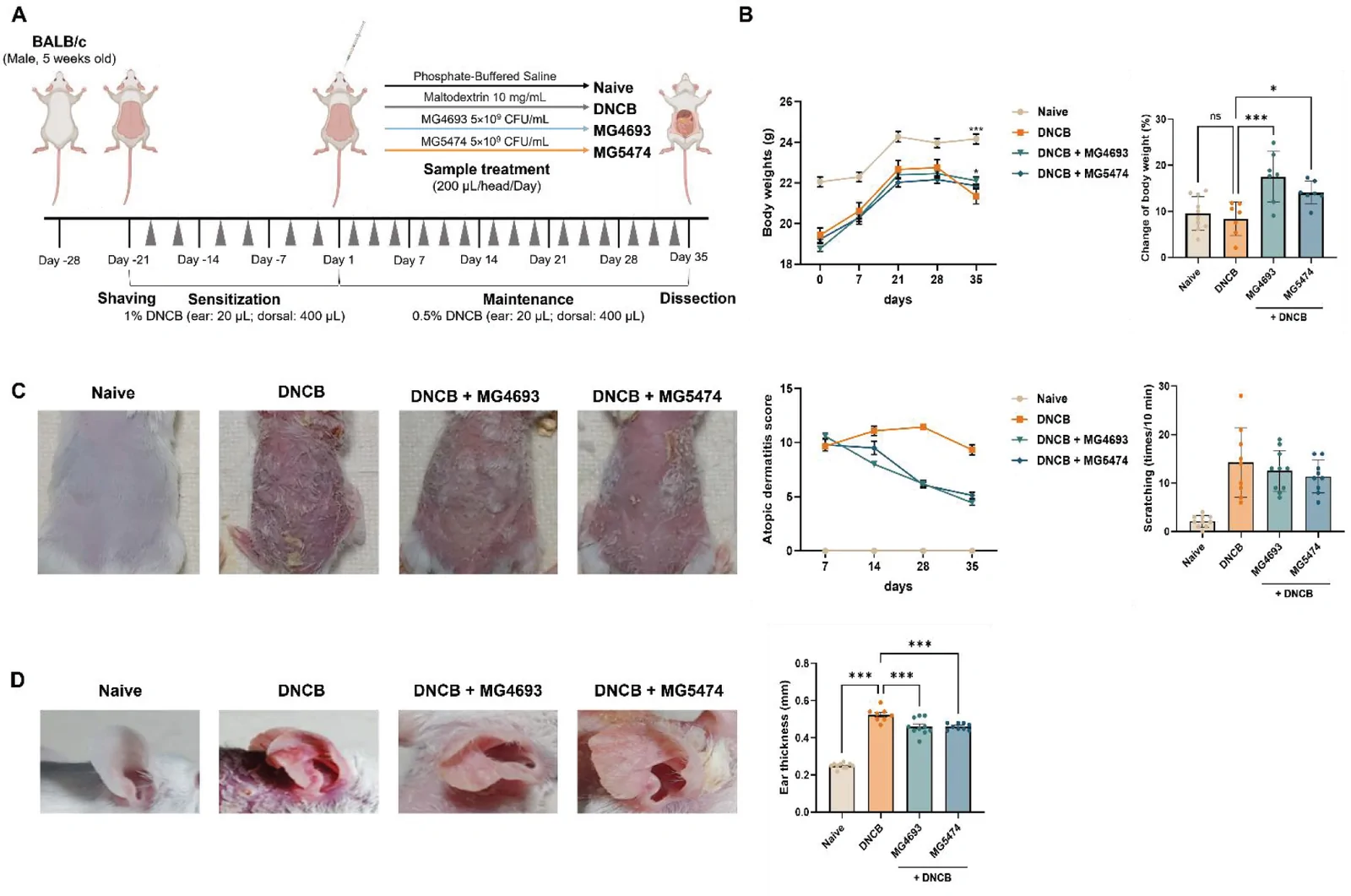
Effects of MG4693 and MG5474 on Atopic Symptom Improvement in AD Mice. (A) Schematic representation illustrating the experimental groups and the administered samples. (B) Body weight changes during the 5-week study period. (C) Representative images of dorsal skin lesions are shown with corresponding dermatitis scores, while scratching frequency was measured over 10-min period. (D) Representative images of mouse ears and corresponding ear thickness measurements. Results are presented as mean ± SD. Significant differences indicate the means at * *p* < 0.05, ** *p* < 0.01, and *** *p* < 0.001 with Dunnett’s multiple comparisons test.

A DNCB-induced AD mouse model was established through 3 weeks of sensitization, followed by 5 weeks of oral administration of MG4693 or MG5474 under continued DNCB challenge (Fig. 1A). Mice in the DNCB group exhibited a significant reduction in body weight compared with the naive group. In contrast, treatment with MG4693 or MG5474 significantly increased body weight relative to the DNCB group (Fig. 1B). Furthermore, to evaluate AD severity, the frequency of scratching and the symptoms of ear swelling and erythema were assessed. A declining tendency in scratching frequency was observed in the probiotic-treated groups; however, no significant differences were observed between groups (Fig. 1C). Pronounced edema and erythema were observed in the ears of AD-induced mice (Fig. 1D). The ear thickness was markedly increased in the DNCB group compared with that of the naive group. In contrast, the MG4693- or MG5474-treated groups showed a significant reduction in ear thickness compared to the DNCB group (*P* < 0.001).

### Reduction of serum IgE and IL-4 levels following MG4693 and MG5474 treatment

**Fig. 2:**
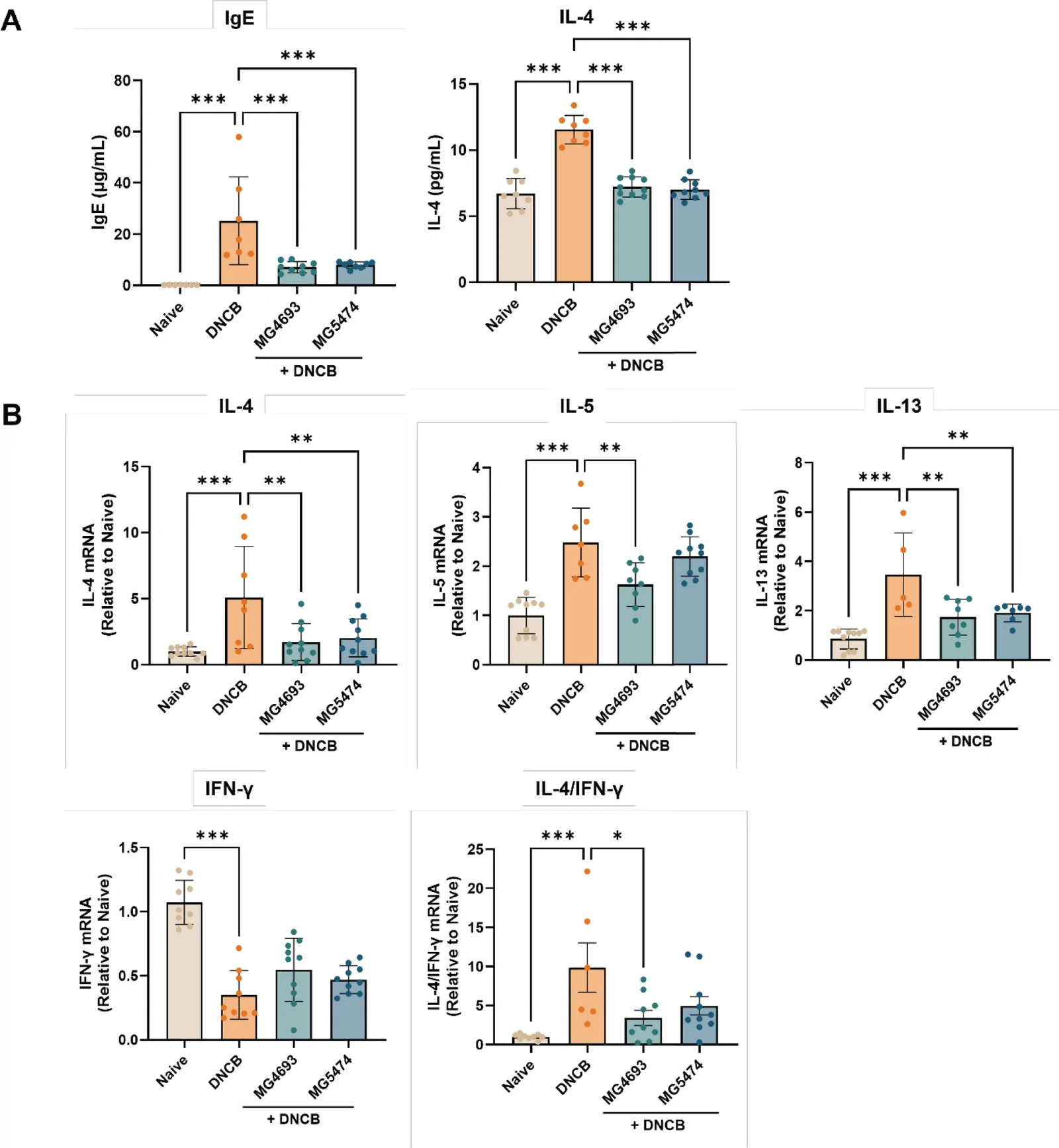
Effects of MG4693 and MG5474 on expression of IgE and T helper cytokines in the serum and dorsal skin tissues of AD mice. (A) Serum IgE and IL-4 levels were measured by ELISA. (B) Cytokine mRNA expression levels were determined by quantitative RT-PCR. IgE, immunoglobulin E; IL-4, interleukin-4; IFN-γ, interferon-γ. Results are presented as mean ± SD. Significant differences indicate the means at * *p* < 0.05, ** *p* < 0.01, and *** *p* < 0.001 with Dunnett’s multiple comparisons test.

Serum IgE and IL-4 levels were significantly higher in the DNCB group than in the naive group (*P* < 0.001). In contrast, treatment with MG4693 or MG5474 significantly reduced these levels compared with the DNCB group (*P* < 0.001) (Fig. 2A).

### Regulation of cytokine and IFN-γ mRNA levels by MG4693 and MG5474 treatment

The Messenger RNA (mRNA) expression levels of Th2-associated cytokines (IL-4, IL-5, and IL-13) in dorsal skin tissues were markedly elevated in the DNCB group compared with the naive group (*P* < 0.001), whereas IFN-γ expression was reduced (Fig. 2B). Treatment with MG4693 or MG5474 significantly reduced IL-4 and IL-13 expression compared with the DNCB group (*P* < 0.01), whereas IL-5 expression declined remarkably only in the MG4693-treated group (*P* < 0.01). IFN-γ expression levels were reduced in the DNCB group compared with the naive group and were not significantly altered by strain treatment. Notably, the IL-4/IFN-γ ratio was significantly decreased in the MG4693 group compared with the DNCB group (*P* < 0.05), suggesting a shift toward a more balanced Th1/Th2 immune response.

### Improvement of dorsal skin histopathology by MG4693 and MG5474 treatment

**Fig. 3:**
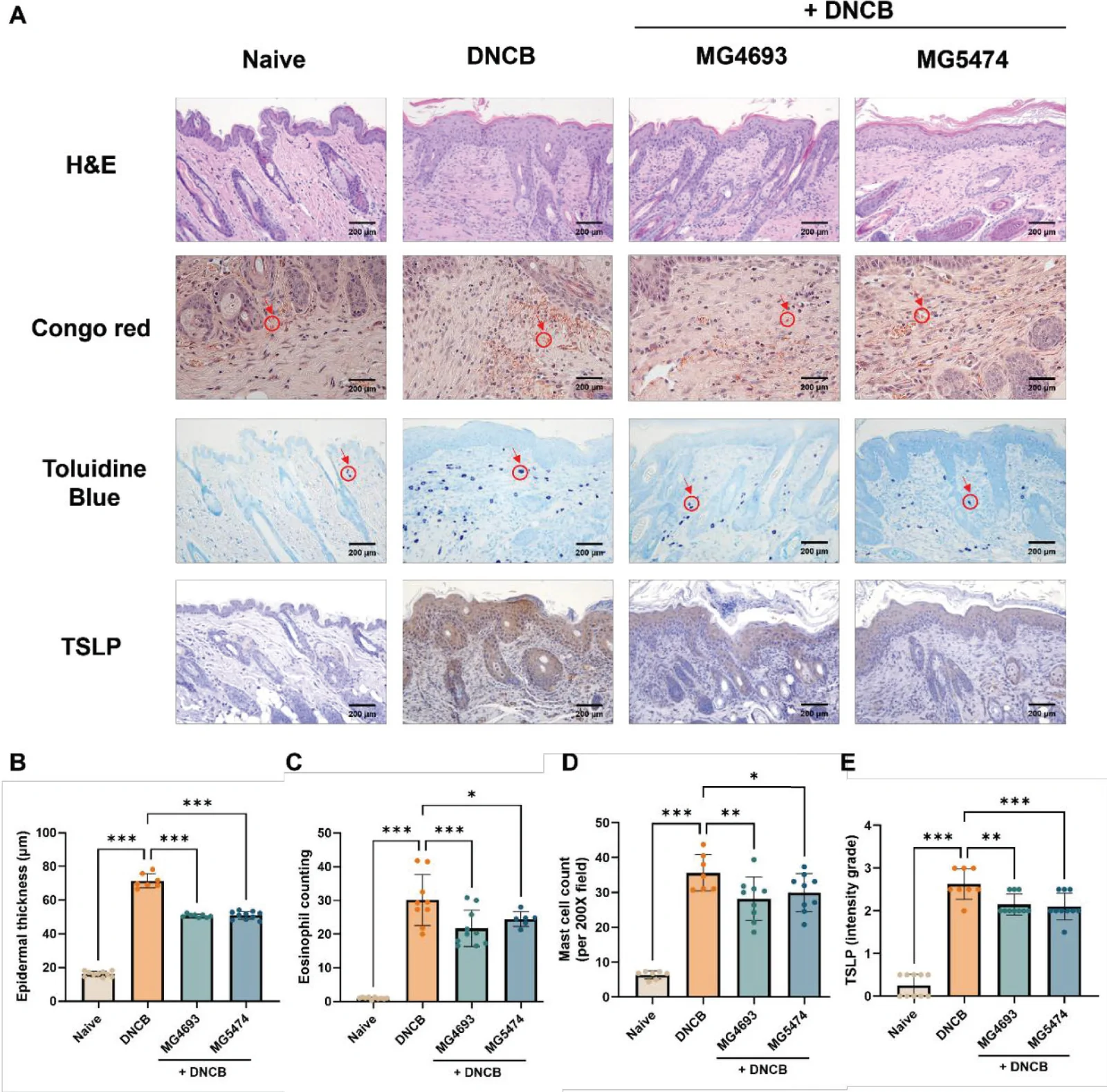
Histological analysis of dorsal skin tissues in AD mice. (A) Representative images of skin sections stained with H&E, Congo red, Toluidine blue, and immunohistochemistry for thymic stromal lymphopoietin (TSLP). Red arrows indicate eosinophils in Congo red and mast cells in Toluidine blue. (B) Quantification of epidermal thickness. (C) Eosinophil counts in skin. (D) Mast cell counts in skin. (E) TSLP expression in skin tissues. TSLP, thymic stromal lymphopoietin. Results are presented as mean ± SD. Significant differences indicate the means at * *p* < 0.05, ** *p* < 0.01, and *** *p* < 0.001 with Dunnett’s multiple comparisons test.

Histological changes in the dorsal skin tissues of DNCB-induced AD mice were evaluated using H&E, Congo red, and toluidine blue staining (Fig. 3A). Epidermal thickness, as measured by H&E staining, was significantly increased in the DNCB group and markedly reduced in the MG4693 or MG5474 group compared with the DNCB group (*P* < 0.001) (Fig. 3B). Inflammatory cell infiltration was further assessed by Congo red staining for eosinophils and toluidine blue staining for mast cells. The number of eosinophils and mast cells was significantly increased in the DNCB group, whereas the cell populations of both were significantly reduced following treatment with MG4693 or MG5474 (eosinophils: MG4693, *P* < 0.001; MG5474, *P* < 0.05; mast cells: MG4693, *P* < 0.01; MG5474, *P* < 0.05) (Fig. 3C and D).

In addition, ICH analysis revealed markedly increased TSLP expression in the dorsal skin of DNCB-induced mice compared with the naive group (*P* < 0.001). Moreover, TSLP expression was considerably reduced in the MG4693 (*P* < 0.01) and MG5474 (*P* < 0.001)-treated groups compared with that of the DNCB group (Fig. 3E).

### Regulation of immune cell populations by MG4693 and MG5474 treatment

**Fig. 4:**
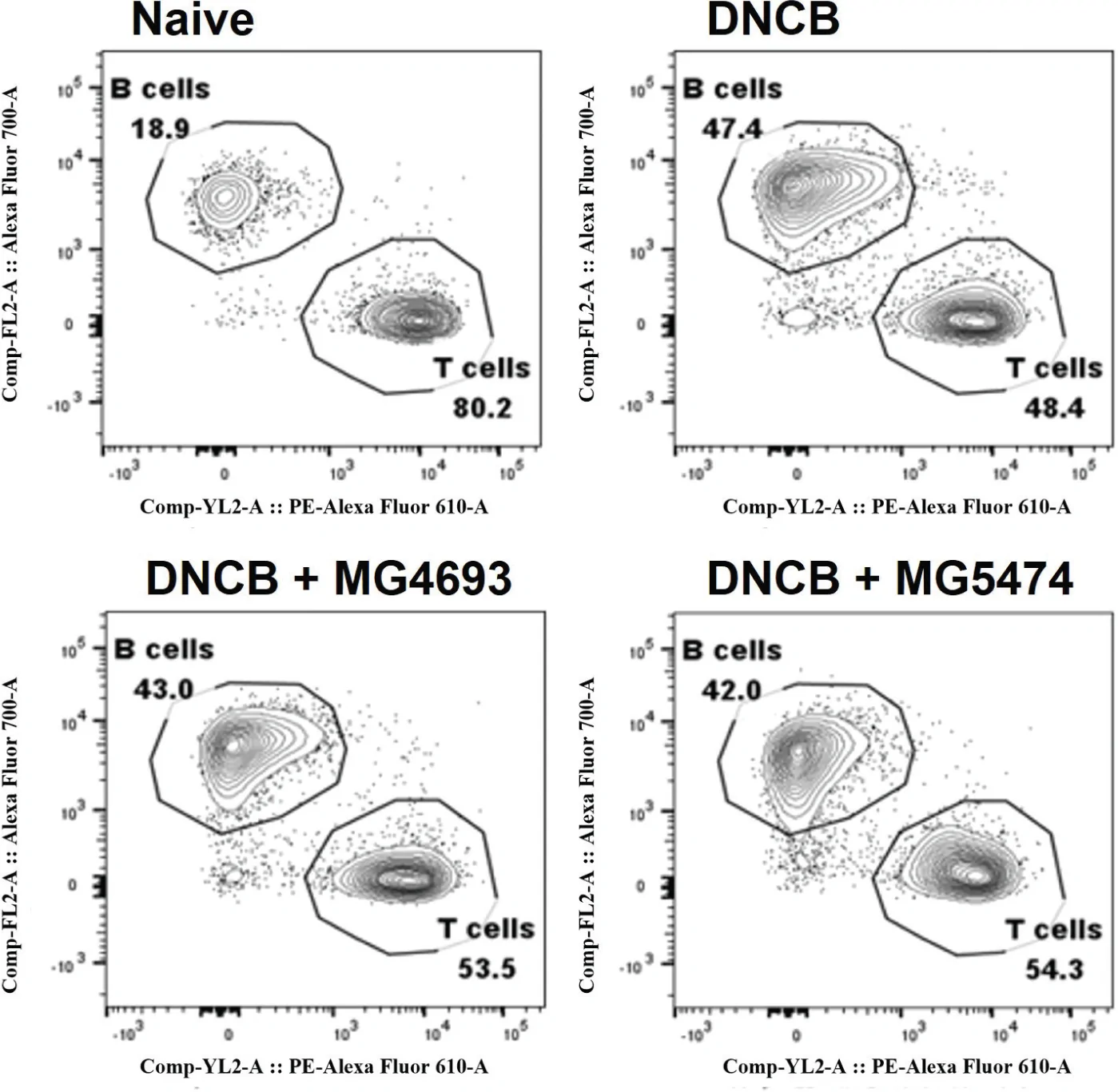
Effects of MG4693 and MG5474 on the expression frequencies of T cell and B cell populations in the lymph nodes of AD mice. Populations in lymph nodes were measured by flow cytometry. (A) Representative flow cytometry plots showing T cells (CD3^+^) and B cells (CD19^+^). (B) Quantification of T cell populations. (C) Quantification of B cell populations. Results are presented as mean ± SD. Significant differences indicate the means at * *p* < 0.05, and *** *p* < 0.001 with Dunnett’s multiple comparisons test.

T- and B-cell populations in the draining lymph nodes were quantified by flow cytometry (Fig. 4A). The number of T-cells in the DNCB group was substantially lower than in the naive group (*P* < 0.001). In contrast, T-cell numbers were significantly increased in the MG5474 group relative to the DNCB group (*P* < 0.05) (Fig. 4B). Conversely, B-cell numbers were markedly elevated in the DNCB group compared with the naive group (*P* < 0.001), whereas MG5474 treatment significantly reduced B-cell numbers compared with the DNCB group (*P* < 0.05) (Fig. 4C), suggesting a partial restoration of immune cell balance in the lymph nodes.

### Intestinal barrier integrity enhancement by MG4693 and MG5474 treatment

**Fig. 5:**
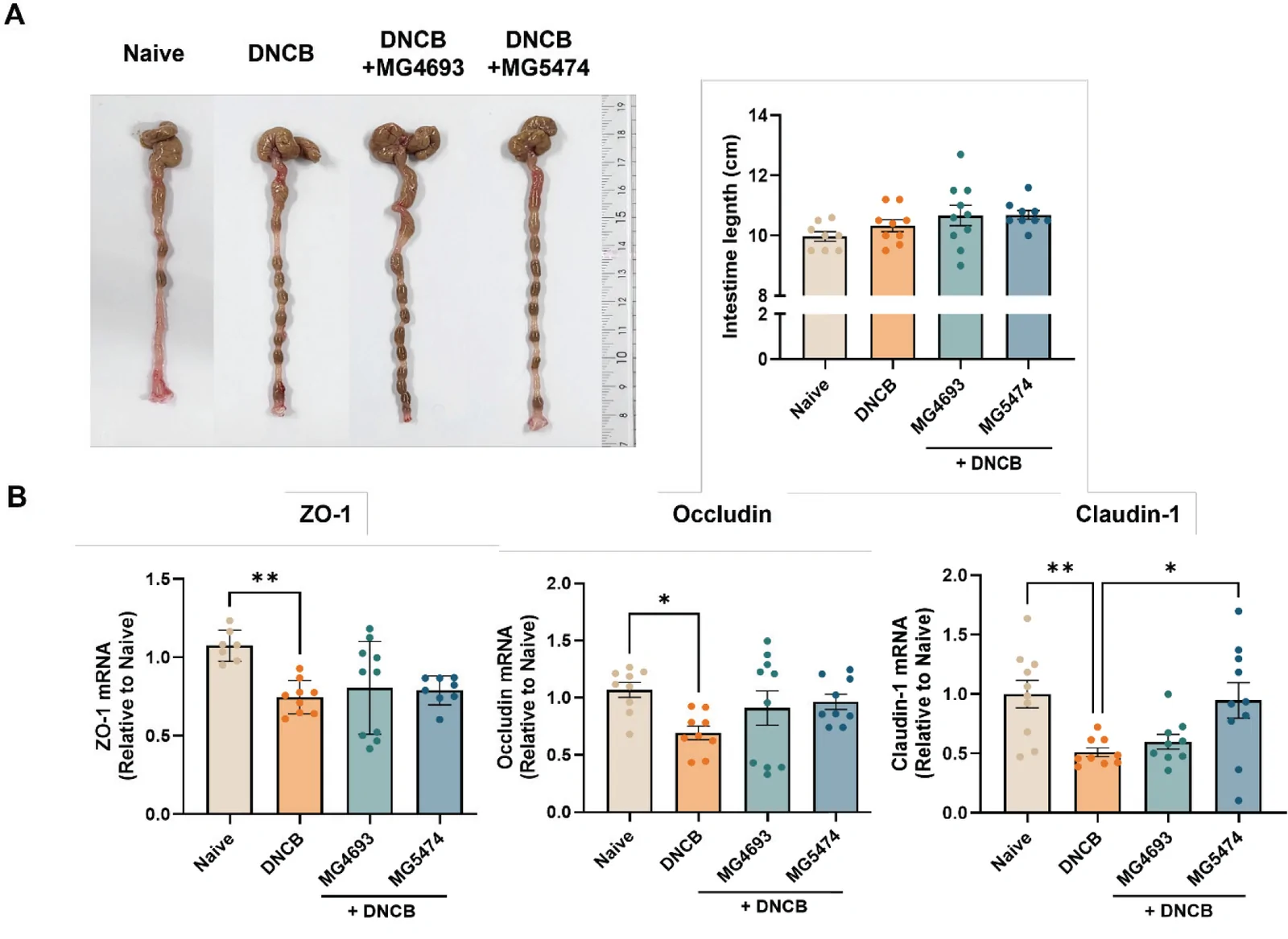
Intestinal morphology and tight junction gene expression in AD mice. (A) Intestinal length was measured in each group. (B) Relative mRNA expression levels of ZO-1, occludin, and claudin-1 in intestinal tissues. Results are presented as mean ± SD. ZO-1, Zonula Occludens-1. Significant differences indicate the means at * *p* < 0.05, and ** *p* < 0.01 with Dunnett’s multiple comparisons test.

To assess intestinal barrier-related changes, the expression levels of tight junction-associated genes were evaluated in intestinal tissues. No significant differences in intestinal lengths were observed among all groups (Fig. 5A). However, the qPCR analysis revealed that the expression levels of tight junction-related genes, including ZO-1, occludin, and claudin-1, were significantly reduced in the DNCB group compared with the naive group. Notably, the treatment of MG5474 significantly increased claudin-1 expression levels compared with the DNCB group (*P* < 0.05) (Fig. 5B).

## Discussion

Recent findings underscore the importance of the gut–skin axis, which links the intestinal microbial composition and skin health (14). Disruption of the gut barrier integrity facilitates the translocation of microbe-derived proinflammatory mediators and metabolites into the systemic circulation, thereby promoting cutaneous inflammation (21). Supplementation with probiotics has been proposed to ameliorate the symptoms of AD, which may be partially mediated by the restoration of intestinal barrier function and regulation of the gut microbiota-based signaling pathways (22). In our previous *in vitro* study, MG4693 and MG5474 suppressed the production of Th2-associated cytokines and chemokines in HaCaT keratinocytes by inhibiting the NF-κB and MAPK signaling pathways (20). Based on their anti-inflammatory efficacy and stability, these probiotic strains were selected for *in vivo* evaluation in an AD mouse model.

Interestingly, mice with AD showed improved suppression of body weight gain with probiotic supplementation. These results are consistent with previous studies on body weight changes in DNCB-induced AD mice, which suggest systemic metabolic involvement (23). Furthermore, representative photomicrographs of the dorsal skin indicated increased erythema, edema, and scaling in AD mice, whereas keratinization and erythema were noticeably reduced in probiotic-treated mice. These marked changes, including suppressed scaling and erythema, closely align with the previously described criteria for AD symptoms (24). Ear thickening in AD mice was accompanied by proinflammatory cell infiltration and epidermal hyperplasia, promoted by barrier destruction (25). Consistently, recent studies have demonstrated marked epidermal hyperplasia, accompanied by increased ear thickness, in DNCB-induced AD mice (26, 27). In addition, elevated scratching in AD mice was mediated by pruritogenic cytokines, including IL-31 and TSLP. Furthermore, recent studies have shown that blocking IL-31-based signaling or reducing macrophage levels suppressed this scratching behavior (28, 29). Probiotic supplementation reduced scratching frequency, thus suggesting a potential mechanism for alleviating pruritus via immunomodulatory effects.

AD is characterized by the overactivation of Th2-derived immunity. This process results in B-cell class switching and increased IgE production (30). Furthermore, IL-4 acts as a central mediator by upregulating IgE and Th2-type proinflammatory responses (31). We observed a marked increase in serum IgE and IL-4 levels in AD mice, which indicates an immune imbalance. Notably, probiotic supplementation significantly reduced the IgE and IL-4 levels. This finding supports previous studies that have shown that probiotics suppress Th2 overactivation and concomitant systemic allergic inflammation (32, 33).

Chronic pruritus in AD fosters a persistent inflammatory response and lichenification of the epidermis, ultimately leading to epidermal thickening (34). Probiotics are a class of therapeutics that inhibit such thickening, further suggesting that they play a role in suppressing symptoms such as inflammatory cell invasion and epidermal hyperplasia. Increased eosinophil invasion was a marker of atopic inflammatory skin disease and correlated with disease severity (35). Building on the role of inflammatory cells in AD, mast cells contribute to AD pathogenesis by releasing histamine and Th2-related cytokines that exacerbate pruritus and inflammation (36). In our study, mast cell invasion in the AD mouse was markedly inhibited by probiotic therapy, which is consistent with other findings that probiotics suppress mast cell and eosinophil invasion in a DNCB-induced AD model (37). Enhanced TSLP expression promotes dendritic cell activation and subsequent Th2 differentiation, producing high levels of IL-4, IL-5, and IL-13 that exacerbate cutaneous inflammation and pruritus (38). Findings demonstrating that TSLP is a major allergenic protein that regulates allergic inflammation by initiating a Th2-type immune response support such a mechanism (39). Enhanced epidermal TSLP expression was observed in our mouse model of AD, whereas probiotic therapy markedly inhibited this activity, suggesting that probiotics can modulate epithelial cytokine signaling to suppress AD pathology. qPCR analysis of dermal skin tissues revealed an elevated expression of Th2 cytokines, including IL-4, IL-5, and IL-13, accompanied by reduced IFN-γ expression. Such a cytokine profile reflects Th2 dominance, a characteristic of AD that drives IgE production, eosinophil recruitment, and tissue inflammation (40). The IL-4/IFN-γ ratio has been proposed as a reliable indicator of Th1/Th2 balance during allergic inflammation (41). The MG4693 group animals showed a significant reduction in this ratio, supporting the ability of probiotics to counteract Th2 dominance and restore immune equilibrium, which is disturbed in AD.

T-cells play a major role in AD pathogenesis, with overactivated Th2 cells secreting IL-4, IL-5, and IL-13 to induce IgE class switching and eosinophil activation (42). B-cells react to Th2 cytokines by changing the IgE class to elevated serum IgE levels; IgE subsequently binds to mast cells and basophils to trigger an allergic response and enhance pruritus and inflammation (43). Flow cytometry revealed that AD induction increased B-cell counts while reducing T-cell proportions in lymphoid tissues. MG5474 partially reversed these changes, suggesting an immunomodulatory effect on the adaptive immune response.

In our previous *in vitro* study, the supernatant derived from lactic acid bacteria suppressed NF-κB activation in tumor necrosis factor-alpha (TNF-α)/IFN-γ-stimulated HaCaT keratinocytes, accompanied by decreased phosphorylation of IκBα and NF-κB (20). NF-κB is a pivotal transcription factor that links cytokine signaling to inflammation-related gene expression, and its activation leads to the production of Th2-related cytokines and chemokines, including TSLP, IL-4, and thymus and activation-regulated chemokine (TARC) (44). Therefore, inhibition of NF-κB signaling by MG4693 and MG5474 may partly contribute to the immunomodulatory effects observed *in vivo*, including reduced B-cell-associated responses and IgE production. Collectively, these results indicate that the anti-inflammatory responses observed *in vivo* are likely mediated, at least in part, by NF-κB-dependent mechanisms operating in keratinocytes.

Patients with AD exhibit increased intestinal permeability, exacerbated intestinal microbial imbalance, and barrier dysfunction, which contribute to the onset of disease (45, 46). Such alterations in the intestinal environment are associated with local immune dysregulation and may also exert systemic consequences via the gut–skin axis (47). Persistent and severe intestinal inflammation induces structural gut alterations, including villus blunting, crypt hyperplasia, and, in extreme cases, intestinal shortening (48). In our AD mouse model, no evident structural changes were observed; however, the expression of tight junction-related genes was reduced but restored by probiotic supplementation, thereby indicating their protective influence on epithelial barrier integrity (49). This absence of histological alterations may be attributable to the relatively short induction and treatment periods, as structural remodeling, including villus blunting or crypt hyperplasia, typically occurs only with prolonged or severe intestinal inflammation (50). Nevertheless, the intestinal barrier-related findings should be interpreted with caution, as this study assessed only mRNA expression of tight junction-associated genes without protein-level validation, such as Western blotting or immunostaining. Therefore, these results provide preliminary evidence supporting the role of intestinal barrier regulation in the gut–skin axis, and further studies are needed to confirm whether these transcriptional changes are accompanied by structural and functional improvement of the intestinal barrier.

The regulation of tight junction-related gene expression by probiotics suggests a potential role in supporting gut epithelial integrity. These changes were accompanied by systemic immunomodulation, including suppression of Th2 cytokines and IgE levels and mast cell- and eosinophil-mediated responses, as well as partial restoration of the Th1/Th2 balance (21). Through such regulation, probiotics ultimately ameliorated cutaneous manifestations of AD, as evidenced by reduced ear and epidermal thickness, decreased scratching behavior, and downregulation of TSLP expression in skin tissues. These findings are consistent with our previous *in vitro* study, in which MG4693 and MG5474 adhered effectively to intestinal epithelial cells and suppressed inflammatory responses in keratinocytes by inhibiting NF-κB signaling (20). Collectively, these findings indicate that probiotics may serve as candidate functional foods for alleviating AD by targeting the gut–skin axis.

## Conclusion

In conclusion, *L. paracasei* MG4693 and *L. lactis* MG5474 alleviated AD by enhancing intestinal barrier integrity and modulating systemic immune responses. Restoration of tight junction-associated gene expression and suppression of Th2 cytokines, IgE-related responses, mast cells, and eosinophils contributed to improvements in skin symptoms. These findings highlight the potential of MG4693 and MG5474 as candidate functional probiotics that mitigate AD by regulating the gut–skin axis.

## Data Availability

Data will be made available on request.
